# Studies on the Effect of Mass Transfer in Vacuum Impregnation on the Bioactive Potential of Apples

**DOI:** 10.3390/molecules24193533

**Published:** 2019-09-29

**Authors:** Marta Pasławska, Bogdan Stępień, Agnieszka Nawirska-Olszańska, Kinga Sala

**Affiliations:** 1 Institute of Agricultural Engineering, Wroclaw University of Environmental and Life Sciences, Chełmońskiego Street 37a, 51-630 Wrocław, Poland; bogdan.stepien@upwr.edu.pl (B.S.); ksala195@gmail.com (K.S.); 2 Department of Fruit, Vegetable and Plant Nutraceutical Technology, Wroclaw University of Environmental and Life Sciences, Chełmońskiego Street 37, 51-630 Wrocław, Poland; agnieszka.nawirska-olszanska@upwr.edu.pl

**Keywords:** vacuum impregnation, mass transfer, apple, polyphenols, antioxidant capacity

## Abstract

The purpose of the study was to evaluate the efficiency of mass transfer during vacuum impregnation (VI) of apple tissue by different process conditions. VI was carried out in two stages: Vacuum (4, 6, or 8 kPa maintained at time 10, 20, 30, 40, 60, and 80 s) and atmospheric (4 min under atmospheric pressure). As infiltration liquids, fresh squeezed apple-pear juice (J), 3% citric acid solution (C), and distilled water (DW) were used. Mass transfer was analyzed based on three factors: Mass variation (MV), dry mass variation (DMV), and solid gain (SG). The outflow of native components and inflow of infiltration liquid has been described by mathematical models. The polyphenol content and antioxidant capacity (ABTS^+^, FRAP) were evaluated as the bioactive potential factors confirming native component outflow and incorporation of liquid molecules into an apple tissue. It was found that during VI of an apple tissue, intensive mass transfer occurred: Native components of fruit tissue outflowed and external ingredients of impregnation liquid inflowed into the material with the intensity proportional to the vacuum level and process time. The most beneficial conditions of apple cube VI were noticed at a vacuum level of 4 kPa for a minimum of 40 s, which is when the highest polyphenol content and antioxidant capacity occurred.

## 1. Introduction

Osmotic dehydration occurs in hypertonic solutions of high osmotic pressure and water activity and is based on the simultaneous two-way diffusion phenomenon: Outflow of tissue water to the solution and inflow of the solution to the tissue [1]. However, while the osmotic dehydration of foodstuffs is one of the simplest and most inexpensive methods of modifying food properties, it seems to be a relatively inefficient and time-consuming process [2]. Due to the fact that the osmotic pressure—as the only driving force for mass transfer—does not provide high processing rates, a number of innovations (vacuum and/or ultrasound treatments) have been studied to improve process efficiency [3].

Vacuum impregnation (VI) is a variation of osmotic dehydration but is carried out in a vacuum, after initial removal of the intercellular air (with some native liquids) from the processed tissue due to the pressure changes [4,5,6]. The emptied intercellular spaces and pores can be easily filled with an impregnation liquid, with the intensity related to the porosity of plant tissue and process conditions: The vacuum level, the time of treatment, and the osmotic pressure. Moreover, the impregnation liquid does not have to be as highly concentrated as during osmotic dehydration, which can be crucial in creating healthy low sugar and low salt foods. VI led to the obtainment of new products of high quality and unique, very beneficial properties, because the active compounds have particular roles in human health (probiotics, vitamins, antioxidants, antimicrobials, or other bioactive compounds), and can be incorporated into the fruit or vegetable during an intensive mass transfer under controlled, non-destructive conditions [7,8,9,10,11]. Low temperature and relatively short time of treatment by VI limits the unfavorable changes in cell structure, color, smell, and taste of food [12,13]. The application of a vacuum at the beginning of osmotic dehydration also increases the water loss and the uptake of osmotic solutes [5,6,14]. For that reason, VI is recommended as the energy-saving pre-treatment before the drying, freezing, or frying of different fruits and vegetables [15,16]. Energy saving effects can be achieved by removing the intercellular water without providing heat, while further water removal during the dehydration process requires less energy than in non-impregnated materials [17].

Control of the VI process requires knowledge of the correlation between the material being treated and the process conditions: the type and temperature of the osmotic solution, the vacuum, the duration of the process, the shape of the sample, and the uniformity of contact of the osmotic solution with the sample [7,18]. Therefore, the objective of this work was to study the mass transfer efficiency during vacuum impregnation of apple cubes by different process conditions. The aim was to develop a procedure to obtain a fruit product enriched with natural apple-pear juice and an antibrowning agent—the citric acid solution.

## 2. Results and Discussion

### 2.1. Mass Variation (MV)

Values of indicators describing mass variation (MV) (Equation (2)) in specific phases of the vacuum impregnation process are presented in Table 1. Depending on process conditions, the sample mass after impregnation differed significantly from raw material mass. If no mass exchange occurred during the impregnation, samples before and after the process would theoretically have an identical mass. However, in reality they differed significantly.

For all impregnating liquids and all vacuum levels, the sample mass increased at each phase of impregnation, starting from the first measurement taken 10 s after the process began. This means that empty intercellular spaces were immediately filled with the infiltration liquid, and that during the process, more ingredients (in terms of mass) of the impregnating solution were taken into the raw material than were removed. Statistical analysis of the influence of impregnation time duration on sample mass value showed that for the majority of test variants, sample mass stabilized as early as 20 s into the process, and longer impregnation did not cause further significant changes. Other researchers have claimed that stabilization of the vacuum impregnation process was achieved after about 50 s [19] or even 100 s [20].

Impregnation kinetics depend very strongly on the type of raw material undergoing impregnation; in the case of impregnation of potatoes with a 10% solution of citric acid, an increase of citric acid content was registered only after 1 h of impregnation [10]. During impregnation of apples with apple-pear juice, sample mass increased from between 0.9 to 4.6% as compared to raw material mass. Increasing the vacuum pressure level in the impregnator chamber caused an increase of impregnated sample mass. The impregnation conducted using citric acid solution resulted in samples with masses from 8.7% to 30% greater than raw material mass depending on the vacuum pressure level. Similar levels of mass increase during vacuum impregnation were achieved by Derossi et al. [21], impregnating black pepper flakes in lactic acid solution, and by Schulze et al. [22], impregnating apples in solutions based on concentrated apple juice. The mass of apple cubes that underwent impregnation with distilled water was higher than the mass of samples impregnated with 3% citric acid solution but lower than the mass of samples impregnated with apple–pear juice.

In the case of 3% citric acid solution and apple-pear juice used as impregnates, a statistically significant influence on the sample mass was noted. The higher the vacuum pressure in the impregnator chamber was, the higher the mass of the samples after the process. Similar dependency was not determined during impregnation of apples with distilled water. It seems, therefore, that the osmotic gradient effect had the biggest influence on sample mass after impregnation.

### 2.2. Dry Mass Variation (DMV)

Testing the vacuum impregnation of apples using distilled water as the impregnating solution was intended to determine the influence of the impregnation mechanism on dry mass content variation. The results were not as positive as we expected after MV analysis (Table 2). Research has shown that impregnating high density solutions limits the possibility of penetration of apple parenchyma tissue by the solutions’ ingredients.

Only for impregnation conducted using apple-pear juice was dry mass content in the material higher after impregnation than in the raw material. Statistically significant differences in dry mass content during specific impregnation phases for products obtained using various impregnating solutions showed an intense saturation of the apple structure with soluble ingredients of apple-pear juice or citric acid, respectively.

The small influence of vacuum pressure level in the impregnator chamber on dry mass content in the product was noted. Generally, dry mass increase in apples impregnated in higher pressure conditions can be observed. However, the differences are, in numerous cases, statistically insignificant.

There is significant influence on the internal structure of the product [9,20] with shortening of time for possible further processing (drying). Such distinct differences between results obtained for a variety of impregnating solutions are probably a consequence of large differences in the density of the liquids used [23]. Intense effluence of valuable cell ingredients from the tissue structure during vacuum impregnation is a side-effect that should be avoided by using as mild impregnation conditions as possible or by prolonging the phase of maintaining the material in the impregnates after retaining atmospheric pressure.

### 2.3. Solid Gain Changes (SG)

In order to describe precisely the phenomenon of the mass transfer in apples vacuum impregnated with three impregnating liquids at various levels of vacuum, solid gain (SG) changes were analyzed using mathematical models frequently recommended for osmotic dehydration: Peleg’s model (Equation (5)), Kelvin-Voigt model (Equation (6)), and Burger’s model (Equation (7)).

#### 2.3.1. VI with Citric Acid Solution

It was noticed that of the three tested mathematical models, the best fitting the VI of apple cubes with citric acid solution was the Kelvin-Voigt model (Table 3).

**Figure 1:**
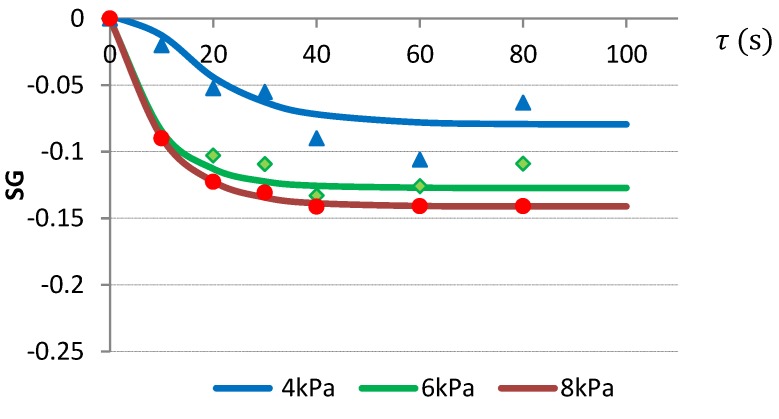
Changes of SG during VI of apples at varied vacuum pressures of 4, 6, and 8 kPa using citric acid solution as the impregnating liquid.

Impregnation with citric acid solution occurred most intensely during the first 30 s of VI, while from the beginning of the impregnation, samples had a greater mass than the raw material (Figure 1). The influence of vacuum level on the mass transfer was noticeable in the form of an increase of SG, the smallest by 4 kPa and the largest by 8 kPa pressure. After 40 s of VI, SG changes stabilized.

#### 2.3.2. VI with Apple-Pear Juice

The best fitting mathematical model of the VI of apple cubes with apple-pear juice was also the Kelvin-Voigt model (Table 4).

**Figure 2:**
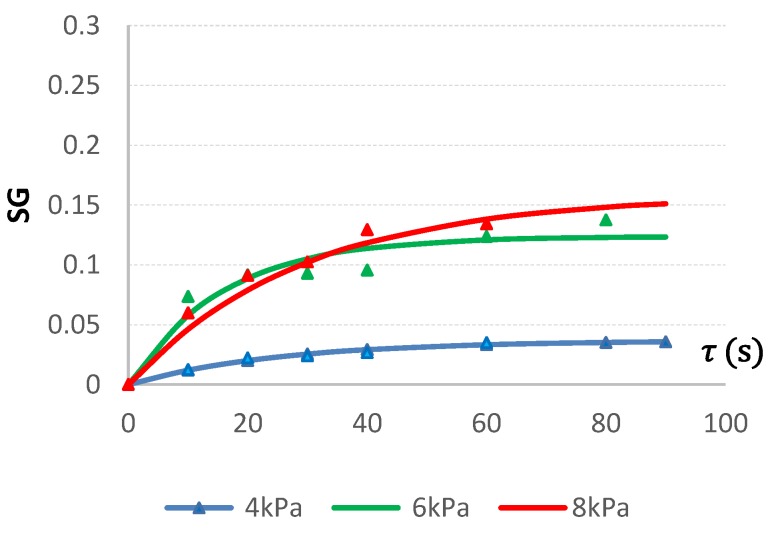
Changes of SG during impregnation of apples at varied vacuum pressures of 4, 6, and 8 kPa using apple-pear juice as the impregnating liquid.

Figure 2 shows SG changes during VI of apples with fresh squeezed fruit juice, by using the Kelvin-Voigt model.

In the case of apple-pear juice as impregnate, an SG coefficient increase was noted during the process, but SG achieved significantly lower values than in case of citric acid solution and the influence of pressure was not as clear. The smallest SG increase occurred during VI at a vacuum pressure of 4 kPa, whereas during the first 30 s of impregnation, the vacuum of 6 kPa was more effective than 8 kPa. After 60 s of the VI process, SG stabilized.

#### 2.3.3. VI with Distilled Water

While all tested mass transfer parameters indicated intensive mass transport during VI of apple issued with citric acid solution and juice, only VI with distilled water showed precisely the real native ingredient outflow. It was noticed that the three tested mathematical models recommended frequently for osmotic dehydration (Equations (5)–(7)) did not satisfactorily fit the data of apples VI with distilled water (Table 5); therefore, another mathematical model, generated by the Table Curve 2D v. 5.01 program was proposed (Equation (1)).

(1) $$Ƴ=a+b\cdot {\left(1-n\right)}^{2}\;,\;where\;\;n=exp\;({\frac{-\left(\tau -d\cdot ln\left(-0.2\right)-c\right)}{d}}^{})$$

**Figure 3:**
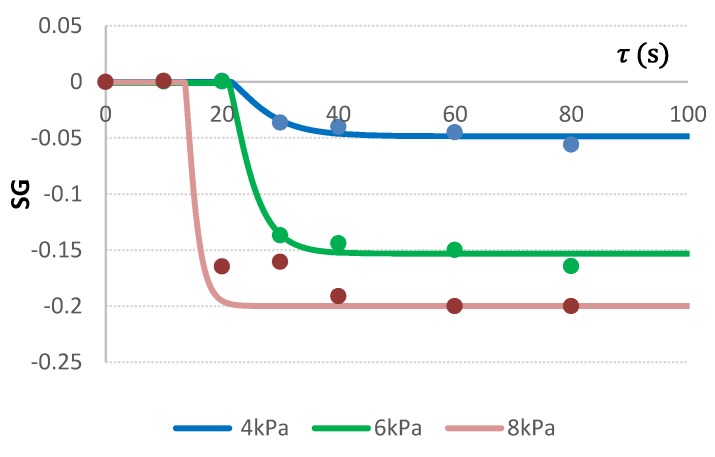
Changes of SG during impregnation of apples at varied vacuum pressures of 4, 6, and 8 kPa using distilled water as the impregnating liquid.

During impregnation of apples with distilled water, negative SG values were noted after 30 s in the case of vacuums 4 and 6 kPa, and after 20 s of process in the case of vacuum 8 kPa. The analysis proved the intense effluence of apple tissue ingredients, increasing proportionally with the vacuum pressure level and duration (Figure 3). After 40 s of impregnation with DW, SG stabilization was achieved.

### 2.4. Polyphenol Content and Antioxidant Activity

**Figure 4:**
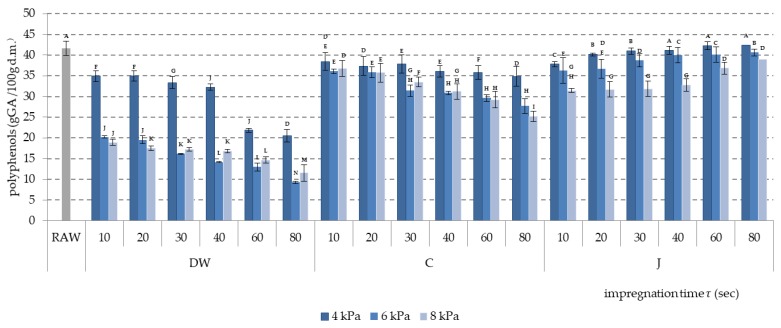
Polyphenol content during vacuum impregnation of apples conducted using apple-pear juice (J), citric acid solution (C), and distilled water (DW) as impregnating liquids. Values followed by different letters (A, B, C…) were significantly different (*p* < 0.05) according to Tukey’s test.

A negative influence on polyphenol content in apple tissue of increasing the level of vacuum pressure during impregnation was noted. In the case of all three impregnating liquids, the lowest polyphenols loss was noted in samples impregnated at a pressure of 4 kPa, and the highest at a pressure of 8 kPa (Figure 4). During impregnation with citric acid solution and distilled water, polyphenol content decreased in time, proving a gradual efflux of polyphenols from apple tissue. This efflux of polyphenols was much more intensive when DW was used, because of a bigger difference in osmotic pressure. When apple-pear juice was used as the impregnating liquid, an increase of polyphenol content with time was noted, meaning that polyphenols lost from the apple cubes were replaced with polyphenols from the apple-pear juice.

**Figure 5:**
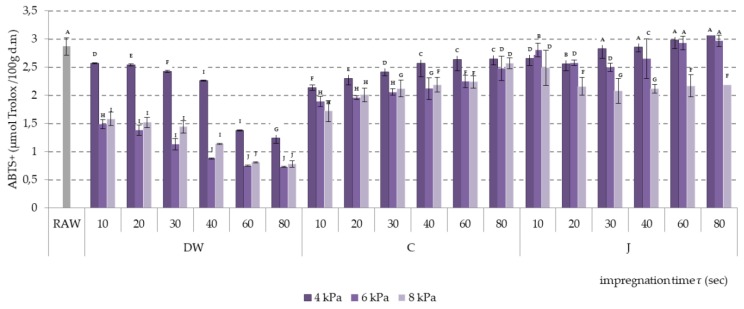
ABTS^+^ antioxidant activity of apples impregnated using vacuum pressures of 4, 6, and 8 kPa and apple-pear juice (J), citric acid solution (C), and distilled water (DW) as impregnating liquids. Values followed by different letters (A, B, C…) were significantly different (*p* < 0.05) according to Tukey’s test.

**Figure 6:**
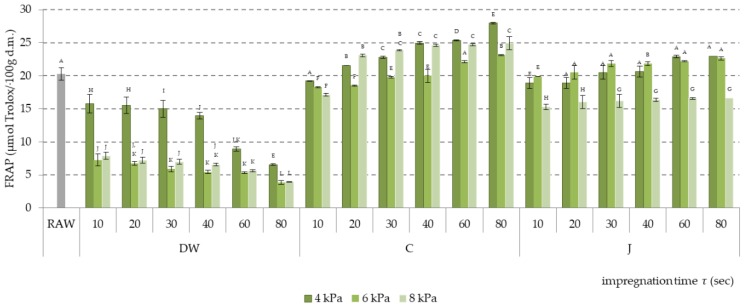
FRAP antioxidant activity of apples impregnated using vacuum pressures of 4, 6, and 8 kPa and apple-pear juice (J), citric acid solution (C), and distilled water (DW) as impregnating liquids. Values followed by different letters (A, B, C…) were significantly different (*p* < 0.05) according to Tukey’s test.

Antioxidant activity in apples impregnated with all three liquids depended on the vacuum pressure level and impregnation time. The highest antioxidant activity was measured for samples impregnated at 4 kPa pressure, both using the ABTS^+^ method and the FRAP method (Figure 5 and Figure 6).

In the case of distilled water and citric acid solution used as impregnates, the antioxidant activity measured with the ABTS^+^ method was lower at 6 kPa pressure than at 8 kPa pressure, with the exception of the initial phase of impregnation with citric acid solution (10 s). The ABTS^+^ antioxidant activity of apples impregnated with apple-pear juice decreased with vacuum pressure level decrease. Only impregnation with apple juice at 4 kPa vacuum pressure enabled increase of antioxidant activity as compared to the raw material.

Influence of vacuum pressure level on antioxidant activity was also noted using the FRAP method (Figure 6). Using 4 kPa pressure allowed high antioxidant activity of impregnated samples to be maintained even in the case of distilled water, when effluence of bioactive ingredients from apples was highest. In the case of other impregnates, influence of pressure on FRAP activity was not as clear. The highest FRAP value was noted for the sample impregnated in citric acid solution at 4 kPa vacuum pressure for 80 s. Impregnation time increased FRAP antioxidant activity above the value determined for raw material from the 40th second in the case of apple-pear juice, and from the 20th second in the case of citric acid solution. This phenomenon confirms the correct progression of impregnation and the effect of building impregnate ingredients into plant tissue.

## 3. Materials and Methods

### 3.1. Apple Preparation

Fresh apples of the *Ligol* variety, with an initial moisture content of 87.3%, were purchased from a local fruit market (trading products from Lower Silesia, Poland) and stored at 4 ± 0.2 °C prior to experiments. Apples, selected and equilibrated at room temperature, were washed, drained with tissue-paper, and cut into cubes of size 10 ± 0.1 mm out of the inner part of the parenchyma.

### 3.2. Impregnation Liquids

Three infiltration liquids were used in the experiment: Fresh squeezed apple-pear juice (J) of 13.2 °Bx, obtained from 10 ± 0.1 kg of apples (*Ligol* variety) and 5 ± 0.1 kg of pears (*Conference* variety) using a juicer (AE 3532 CTC Clatronic, Warsaw, Poland); 3% citric acid solution (C) of 2.4 °Bx (Applichem GmbH, Darmstadt, Germany); and distilled water (DW) as reference. The refractive index was measured using an Atago Digital Brix Refractometer PAL-3 (Atago Co., Ltd., Tokyo, Japan).

### 3.3. Vacuum Impregnation

Vacuum impregnation on an experimental stage VI-2016-MSP (patent pending) was carried out at the Institute of Agricultural Engineering, Wrocław University of Environmental and Life Sciences (Wrocław, Poland), using a vacuum vessel of volume 5.0 dm^3^ combined with a vacuum pump [24]. Samples of 150 g of apple cubes were put into the impregnation vessel and when a vacuum pressure of 4, 6, or 8 kPa was reached (10 s), the impregnation solution was applied under vacuum for 10, 20, 30, 40, 60, or 80 s. The mass ratio of apples to solution was 1:4 [25,26]. The atmospheric pressure was restored (10 s) leaving samples immersed in the liquids for a period of 4 min. The samples were then drained with tissue-paper, weighed, and analyzed. Each treatment was performed in triplicate.

### 3.4. Mass Transfer Phenomenon

The mass transfer phenomenon was analyzed on the basis of mass and dry matter content measurements, after calculating mass variation (MV), dry mass variation (DMV), and solid gain (SG) as follows:

(2) $$MV=\;\frac{M_{\tau }}{M_{0}},$$

(3) $$DMV=\;\frac{m_{\tau }}{m_{0}},$$

(4) $$SG\;=\;\frac{m_{\tau }-m_{0}}{M_{0}}.$$

The dry matter content was determined gravimetrically by drying the sample in a vacuum oven at 70 °C until a constant weight was achieved [27].

Solid gain SG (Equation (4)) was presented as g·g^−1^ of fresh material, and the data were analyzed using three mathematical models commonly used for describing mass transfer during osmotic dehydration [28]: The Peleg (Equation (5)), the Kelvin-Voigt (Equation (6)) or the Burger model (Equation (7)), according the formulas:

(5) $$Ƴ={Ƴ}_{0}\pm \;\frac{\tau }{\left(a_{1}+b_{2}\cdot \tau \right)},$$

(6) $$Ƴ=\;a\cdot \left(1-e^{-b\cdot \tau }\right),$$

(7) $$Ƴ=\;a\cdot \left(1-e^{-b\cdot \tau }\right)+c\cdot \tau .$$

### 3.5. Chemical Characteristics

The fresh and impregnated apples were analyzed according to total polyphenols and antioxidant capacity as well.

Polyphenols were evaluated by the Folin-Ciocalteu method, in which gallic acid is utilized. The sample absorbance was measured at a wavelength of 765 nm (Shimadzu UV-2401 PC spectrophotometer, Osaka, Japan). The result was expressed as a content of gallic acid (mg gallic acid/100 g dry matter) [29].

The antioxidant capacity was tested using the ABTS^+^ [30] and FRAP methods [31].

ABTS^+^ solution was diluted with redistilled water until it reached 0.700 absorbance at a wavelength of 743 nm (Shimadzu UV-2401 PC spectrophotometer, Osaka, Japan), and 60 μL of supernatant solution was then added to 3 mL of ABTS^+^. The absorbance was measured after 6 min.

The reducing potential of apples was determined using the ferric reducing antioxidant power (FRAP) assay. The supernatant (0.3 mL) and FRAP reagent (3 mL) were added to each of them and mixed thoroughly. Trolox was used as a standard. Absorbance was measured at 593 nm (Shimadzu UV-2401 PC spectrophotometer, Osaka, Japan) after 10 min.

Measurements of polyphenols and antioxidant capacity were performed in triplicate.

### 3.6. Statistical Analysis

A one-way ANOVA was performed at the significance level *p* < 0.05, and Tukey’s test was used for multiple comparison. Significant differences (*p* ≤ 0.05) between the mean values of chemical characteristics were determined by Tukey’s multiple range test. The data were recorded as means ± SD (standard deviation), and analyzed using Excel 2007.

Fitting of the mathematical functions (Peleg, Kelvin-Voigt, and Burger) to the experimental points was done using Table Curve 2D v. 5.01 (SYSTAT Software Inc., Chicago, IL, USA).

The degree of adaptation of the mathematical model to the mass transfer was based on the simultaneous analysis of the determined values of: Root mean square error (RMSE), reduced test value (χ^2^), residual variance coefficient (CRV), and the determination coefficient (*R*^2^). These factors were calculated based on the following formulas:

(8) $$RSME=\;\sqrt{\frac{\sum_{i=1}^{N}{\left(MR_{i,p}-MR_{i,e}\right)}^{2}}{N}},$$

(9) $$\chi^{2}=\frac{\sum_{i=1}^{N}{\left(MR_{i,p}-MR_{i,e}\right)}^{2}}{N-n},$$

(10) $$\mathrm{CRV}=\frac{\sqrt{\chi^{2}}}{Y}\;\times 100\%,$$

(11) $$R^{2}=\frac{SS_{M}}{SS_{T}}=\;\frac{\sum_{i=1}^{N}{\left(MR_{i,p}-\;MR_{p}\right)}^{2}}{\sum_{i=1}^{n}{\left(MR_{i,e}-MR_{p}\right)}^{2}}{\;}^{\;}$$

The high value of *R*^2^ and the low values of $\chi^{2}$ and RMSE indicated good fitting of the model to the experimental data. The values of CRV of less than 20% indicated that the model can be used for process predictions.

## 4. Conclusions

During the vacuum impregnation of apples, intense mass exchange between raw material tissue and the impregnating solution was noted. Particularly surprising was the intense effluence of soluble ingredients of raw material dry mass, confirmed during impregnation with distilled water, as a decrease of solid gain, polyphenol content, and antioxidant activity. The vacuum pressure level in the impregnator chamber, together with the type of impregnating solution significantly influenced all examined parameters describing the kinetics of vacuum impregnation of apples and content of compounds deciding material bioactivity. The mass transfer intensity increased proportionally to the vacuum level, which seems to be beneficial in the context of bioactive component incorporation possibilities, but negative in the context of native apple ingredient effluence. VI carried out with low concentrations of citric acid solution caused more intensive outflow of inert liquids than VI with apple–pear juice, which resulted in dehydration of the material (a positive effect when carried out as pretreatment before drying), but also in a decrease in bioactive apple potential (a negative effect).

Due to the possibility of obtaining impregnated apple cubes with a relatively high native ingredient content, the most beneficial vacuum level is 4 kPa with a minimum process time of 40 s.

## Figures and Tables

**Table 1:** Mass variation (MV) during vacuum impregnation (VI) of apples at varied vacuum pressures of 4, 6, and 8 kPa using citric acid solution (C), apple-pear juice (J), and distilled water (DW) as the impregnating liquids.

Impregnation Liquid	Time (s)	Vacuum Level (kPa)		
		4	6	8
C	0	1	1	1
	10	1.087 ± 0.007 ^D^	1.153 ± 0.012 ^E,F^	1.262 ± 0.012 ^D,E^
	20	1.095 ± 0.008 ^D^	1.162 ± 0.016 ^F^	1.297 ± 0.012 ^E^
	30	1.099 ± 0.009 ^D^	1.183 ± 0.019 ^F^	1.292 ± 0.013 ^E^
	40	1.099 ± 0.010 ^D^	1.163 ± 0.014 ^F^	1.294 ± 0.015 ^E^
	60	1.108 ± 0.011 ^D^	1.176 ± 0.020 ^F^	1.300 ± 0.014 ^E^
	80	1.122 ± 0.008 ^D,E^	1.163 ± 0.018 ^F^	1.300 ± 0.011 ^E^
J	0	1	1	1
	10	1.027 ± 0.005 ^B^	1.031 ± 0.002 ^B^	1.041 ± 0.005 ^B,C^
	20	1.016 ± 0.004 ^A^	1.035 ± 0.004 ^B^	1.020 ± 0.005 ^A^
	30	1.016 ± 0.005 ^A^	1.033 ± 0.003 ^B^	1.034 ± 0.004 ^B^
	40	1.009 ± 0.003 ^A^	1.042 ± 0.004 ^B,C^	1.045 ± 0.004 ^C^
	60	1.018 ± 0.004 ^A^	1.040 ± 0.004 ^B,C^	1.046 ± 0.005 ^C^
	80	-	1.025 ± 0.004 ^A,B^	-
DW	0	1	1	1
	10	1.104 ± 0.009 ^D^	1.119 ± 0.015 ^D^	1.138 ± 0.014 ^E^
	20	1.113 ± 0.011 ^D^	1.099 ± 0.022 ^D^	1.094 ± 0.012 ^D^
	30	1.122 ± 0.010 ^D^	1.062 ± 0.012 ^C^	1.110 ± 0.011 ^D^
	40	1.102 ± 0.007 ^D^	1.052 ± 0.010 ^C^	1.086 ± 0.010 ^D^
	60	1.107 ± 0.009 ^D^	1.098 ± 0.017 ^D^	1.078 ± 0.008 ^C,D^
	80	1.087 ± 0.011 ^D^	1.093 ± 0.013 ^D^	1.085 ± 0.011 ^D^

Values are mean ± standard deviation, *n* = 3; in columns, values followed by different letters (A, B, C…) were significantly different (*p* < 0.05) according to Tukey’s test.

**Table 2:** Dry mass variation (DMV) during VI of apples at varied vacuum pressures of 4, 6, and 8 kPa using citric acid solution (C), apple-pear juice (J), and distilled water (DW) as the impregnating liquids.

Impregnation Liquid	Time (s)	Vacuum Level (kPa)		
		4	6	8
C	0	1	1	1
	10	0.944 ± 0.020 ^F^	0.993 ± 0.017 ^F^	1.005 ± 0.022 ^F,G^
	20	0.930 ± 0.022 ^F^	0.977 ± 0.021 ^F^	0.958 ± 0.018 ^F^
	30	0.864 ± 0.019 ^E^	0.951 ± 0.017 ^F^	0.903 ± 0.023 ^E,F^
	40	0.818 ± 0.015 ^D^	0.863 ± 0.025 ^E^	0.900 ± 0.019 ^E,F^
	60	0.746 ± 0.011 ^C,D^	0.732 ± 0.011 ^C,D^	0.885± 0.021 ^E^
	80	0.723 ± 0.008 ^C^	0.815 ± 0.017 ^D^	0.786 ± 0.017 ^D^
J	0	1	1	1
	10	1.012 ± 0.019 ^F,G^	1.081 ± 0.024 ^G,H^	1.065 ± 0.028 ^G^
	20	1.061 ± 0.019 ^G^	1.095 ± 0.023 ^G,H^	1.068 ± 0.033 ^G^
	30	1.074 ± 0.020 ^G^	1.108 ± 0.019 ^H^	1.148 ± 0.026 ^I^
	40	1.079 ± 0.015 ^G^	1.105 ± 0.022 ^H^	1.158 ± 0.027 ^I^
	60	1.089 ± 0.019 ^G^	1.152 ± 0.023 ^I^	1.161 ± 0.025 ^I^
	80	-	1.160 ± 0.029 ^I^	-
DW	0	1	1	1
	10	0.455 ± 0.006 ^A^	0.581 ± 0.011 ^B,C^	0.512 ± 0.007 ^B^
	20	0.710 ± 0.011 ^C^	0.679 ± 0.013 ^C^	0.555 ± 0.006 ^B^
	30	0.713 ± 0.009 ^C^	0.679 ± 0.015 ^C^	0.562 ± 0.008 ^B^
	40	0.716 ± 0.08 ^C^	0.693 ± 0.016 ^C^	0.569 ± 0.006 ^B^
	60	0.785 ± 0.020 ^D^	0.714 ± 0.012 ^C^	0.715 ± 0.011 ^C^
	80	0.810 ± 0.022 ^D^	0.764 ± 0.014 ^C^	0.743 ± 0.009 ^C,D^

Values are mean ± standard deviation, *n* = 3; in columns, values followed by different letters (A, B, C…) were significantly different (*p* < 0.05) according to Tukey’s test.

**Table 3:** Model coefficients and statistical factors in mathematical models fitting the experimental data of solid gain (SG) changes during VI of apples at varied vacuum pressures of 4, 6, and 8 kPa using citric acid solution as the impregnating liquid.

Mathematical Model	Coefficient	Vacuum Level [kPa]		
		4	6	8
Peleg	*a*	−219.1333	−36.2405	−12.3447
	*b*	8.4564	7.2476	6.3128
	*RSME*	0.6679	0.7783	0.9904
	$\chi^{2}$	0.000451	0.000551	0.000026
	*CRV*	38.9	38.9	23.6
	*R* ^2^	0.7786	0.8521	0.9936
Kevin-Voight	*a*	−0.0898	−0.1272	−0.1412
	*b*	23.0830	9.1691	9.9627
	*RSME*	0.0779	0.0796	0.0998
	$\chi^{2}$	0.000398	0.000508	0.000005
	*CRV*	5.6	12.1	8.9
	*R* ^2^	0.8049	0.8638	0.9988
Burger	*a*	−3.1239	−0.2255	−0.1404
	*b*	213.6435	20.4573	9.8616
	*c*	−0.0113	−0.0014	0.0001
	*RSME*	0.7944	0.7955	0.9976
	$\chi^{2}$	0.000279	0.000509	0.000007
	*CRV*	42.0	40.0	27.5
	*R* ^2^	0.8972	0.8977	0.9988

**Table 4:** Statistical factors in mathematical models fitting the experimental data of SG changes during VI of apples at varied vacuum pressures of 4, 6, and 8 kPa using apple-pear juice as the impregnating liquid.

Mathematical Model	Coefficient	Vacuum Level [kPa]		
		4	6	8
Peleg	*a*	94.7765	88.1274	62.4510
	*b*	19.8800	6.8219	4.4803
	*RSME*	0.9724	0.9293	0.9849
	$\chi^{2}$	0.000003	0.000107	0.000113
	*CRV*	45.7	48.2	39.1
	*R* ^2^	0.9834	0.9528	0.9910
Kevin-Voight	*a*	0.0368	0.1237	0.1581
	*b*	25.4490	15.8917	28.9365
	*RSME*	0.0969	0.0879	0.0984
	$\chi^{2}$	0.000004	0.000238	0.000118
	*CRV*	2.4	7.0	2.6
	*R* ^2^	0.9781	0.9193	0.9903
Burger	*a*	0.0157	0.0652	0.1198
	*b*	9.2438	1.2598	16.4327
	*c*	0.0003	0.0009	0.0003
	*RSME*	0.9733	0.9799	0.9766
	$\chi^{2}$	0.000003	0.000005	0.000005
	*CRV*	26.6	43.8	28.5
	*R* ^2^	0.9893	0.9900	0.9906

**Table 5:** Statistical factors in mathematical models fitting the experimental data of SG changes during VI of apples at varied vacuum pressures of 4, 6, and 8 kPa using distilled water as the impregnating liquid.

Mathematical Model	Coefficient	Vacuum Level [kPa]		
		4	6	8
Peleg	*a*	−1315.1964	−330.0180	−110.3170
	*b*	−0.4957	−1.3744	−3.1288
	*RSME*	0.0142	0.0545	0.0473
	$\chi^{2}$	0.005756	0.006651	0.001980
	*CRV*	60.3	69.1	38.8
	*R* ^2^	0.7664	0.6814	0.7935
Kevin-Voight	*a*	−0.9334	−0.3738	−0.2266
	*b*	222.4920	124.4170	26.9840
	*RSME*	0.0142	0.0546	0.0453
	$\chi^{2}$	0.002298	0.002340	0.002900
	*CRV*	60.3	68.9	35.3
	*R* ^2^	0.7664	0.6827	0.8105
Burger	*a*	−0.0348	−0.0209	−4.2633
	*b*	322.9368	217.0000	177.4677
	*c*	−0.0006	−0.0024	−0.0169
	*RSME*	0.0159	0.0618	0.0478
	$\chi^{2}$	0.009879	0.009997	0.008988
	*CRV*	67.4	78.2	37.2
	*R* ^2^	0.7664	0.6736	0.8313
Equation 1	*a*	0.0041	0.0138	0.0169
	*b*	−0.0536	−0.1670	−0.2169
	*c*	26.3255	24.1722	15.0000
	*d*	5.2663	3.3950	1.5217
	*RSME*	0.0071	0.0182	0.0166
	$\chi^{2}$	0.00010	0.00056	0.00013
	*CRV*	14.3	14.7	12.6
	*R* ^2^	0.9650	0.9788	0.9845

## Nomenclature

VI	Vacuum impregnation
C	Citric acid solution
DMV	Dry mass variation (-)
DW	Distilled water
J	Apple-pear juice
MV	Mass variation (-)
*SG*	Solid gain (g/g i.d.m.)
*M*_0_	Initial mass of sample (g)
*M_τ_*	Mass of sample after impregnation (g)
*m*_0_	Initial dry matter of sample (g)
*mτ*	Dry matter of sample after impregnation (g)
τ	Time of vacuum impregnation phase (s)
*a, b, c, d, f*	Parameters of SG model (Equations (1),(5)–(7)) (kg/kg)
Υ	Mean of experimental value of SG (Equations (5)–(7))
MR_i,p_	Predicted value of SG
MR_i,e_	Experimental value of SG
N	Number of observations
n	Number of constants in model equation
S_e_	Standard deviation of the residual component
Y_t_	Arithmetic average of the y variable
SS_M_	Sum of squares for the model
SS_T_	Total sum of squares
y_r_	Actual value of the dependent variable (measured)
y_t_	Expected value of the dependent variable (based on the regression model)
y_ś_	Arithmetic average of the actual dependent variable
RMSE	Root mean square error
χ^2^	Test reduction coefficient
V_e_	Residual variance coefficient
R^2^	Determination coefficient
Subscripts	
0	Initial
τ	Time (s)
A, B, C…	Indicate significant differences
